# Blood and Plasma Volumetric Absorptive Microsampling (VAMS) Coupled to LC-MS/MS for the Forensic Assessment of Cocaine Consumption

**DOI:** 10.3390/molecules25051046

**Published:** 2020-02-26

**Authors:** Roberto Mandrioli, Laura Mercolini, Michele Protti

**Affiliations:** 1 Department for Life Quality Studies (QuVi), Alma Mater Studiorum - University of Bologna, Corso d’Augusto 237, 47921 Rimini, Italy; 2 Research group of Pharmaco-Toxicological Analysis (PTA Lab), Department of Pharmacy and Biotechnology (FaBiT), Alma Mater Studiorum—University of Bologna, Via Belmeloro 6, 40126 Bologna Italy

**Keywords:** microsampling, VAMS, LC-MS/MS, cocaine, benzoylecgonine, ecgonine methyl ester, cocaethylene, whole blood, plasma

## Abstract

Reliable, feasible analytical methods are needed for forensic and anti-doping testing of cocaine and its most important metabolites, benzoylecgonine, ecgonine methyl ester, and cocaethylene (the active metabolite formed in the presence of ethanol). An innovative workflow is presented here, using minute amounts of dried blood or plasma obtained by volumetric absorptive microsampling (VAMS), followed by miniaturized pretreatment by dispersive pipette extraction (DPX) and LC-MS/MS analysis. After sampling 20 µL of blood or plasma with a VAMS device, the sample was dried, extracted, and loaded onto a DPX tip. The DPX pretreatment lasted less than one minute and after elution with methanol the sample was directly injected into the LC-MS/MS system. The chromatographic analysis was carried out on a C8 column, using a mobile phase containing aqueous formic acid and acetonitrile. Good extraction yield (> 85%), precision (relative standard deviation, RSD < 6.0%) and matrix effect (< 12%) values were obtained. Analyte stability was outstanding (recovery > 85% after 2 months at room temperature). The method was successfully applied to real blood and plasma VAMS, with results in very good agreement with those of fluid samples. The method seems suitable for the monitoring of concomitant cocaine and ethanol use by means of plasma or blood VAMS testing.

## 1. Introduction

**Figure 1:**
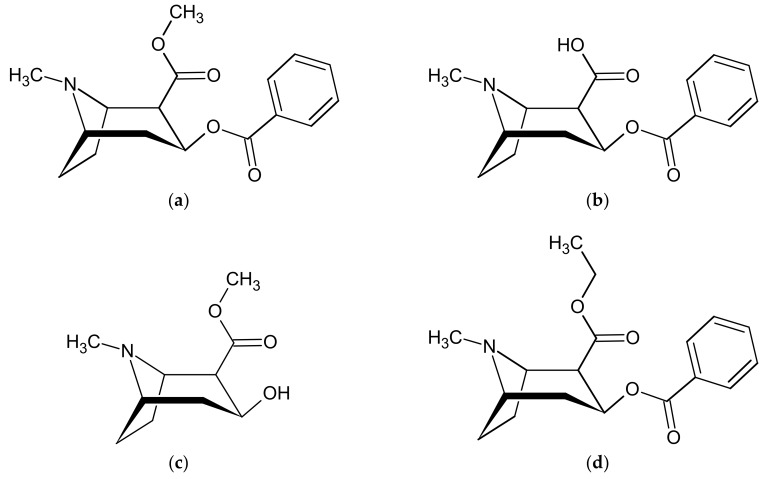
Chemical structures of (**a**) cocaine (COC); (**b**) benzoylecgonine (BEG); (**c**) ecgonine methyl ester (EME); (**d**) cocaethylene (CET).

Cocaine (methyl (1*R*,2*R*,3*S*,5*S*)-3-benzoyloxy-8-methyl-8-azabicyclo[3.2.1]octane-2-carboxylate, Figure 1a, COC) is a natural psychoactive alkaloid extracted from the leaves of the *Erythroxylum coca* plant. It is considered one of the most frequently consumed drugs of abuse, with more than 15 million people currently using this substance worldwide, and with the highest prevalence reaching 2.4% in England and Wales [1]. Despite the recent, dramatic rise in synthetic and natural opioid abuse in the United States (the so called “opioid crisis”), COC use is still much more widespread there than that of opiates (2.1% vs. 0.7% prevalence) and is in the same order of magnitude as total opioids (opiates plus prescription opioids) [2].

Recreational COC consumption can result in several kinds of short- and long-term health damage, as well as psychological and physical dependence, and addiction. For example, exhaustion, somnolence, irritability, and judgment impairment are typical short-term effects, while stroke or ictus, paranoid psychosis and mucosal necrosis can appear over the long term [3,4]. For these reasons, COC is included in the lists of controlled substances of most States [5,6]. In particular, driving while under its influence (DUI) can be very dangerous and is strictly prohibited in most countries, with severe administrative penalties; it is considered a serious aggravating circumstance in the case of car accident [7]. Moreover, COC is included in the World Anti-Doping Agency (WADA) Prohibited List, under Section S6 (stimulants prohibited in-competition) [8].

After consumption, COC is biotransformed by esterases, and partly undergoes spontaneous hydrolysis, to benzoylecgonine ((1*R*,2*R*,3*S*,5*S*)-3-benzoyloxy-8-methyl-8-azabicyclo[3.2.1]octane-2- carboxylic acid, Figure 1b, BEG) and ecgonine methyl ester (methyl (1*R*,2*R*,3*S*,5*S*)-3-hydroxy-8- methyl-8-azabicyclo[3.2.1]octane-2-carboxylate, Figure 1c, EME), which are its main long-term, inactive metabolites [9,10]. When COC and ethanol are simultaneously present in the blood, COC biotransformation is diverted in part to transesterification with the formation of cocaethylene (ethyl (1*R*,2*R*,3*S*,5*S*)-3-benzoyloxy-8-methyl-8-azabicyclo[3.2.1]octane-2-carboxylate, Figure 1d, CET). In contrast to BEG and EME, CET is a psychoactive, stimulant agent with a potency similar to or higher than COC itself and much longer half-life and effect duration, but also with higher cardiotoxicity [11].

As a consequence, reliable and feasible analytical methods are needed to qualitatively and quantitatively assess COC consumption for different purposes: anti-doping, DUI or workplace testing; or in general for forensic analyses in pre- or post-mortem specimens. Of course, these methods should include the determination not only of COC, but also of BEG, EME, and CET, in order to assure maximum detectability of its use [12]. Currently, urine testing is among the most frequent procedures, as it is not invasive, but can be prone to sample adulteration and a positive urine test cannot be considered conclusive proof of actual intoxication state at the moment of sampling.

In the following, a new, original method is described, based on blood or plasma volumetric absorptive microsampling (VAMS) followed by ultrasound-assisted extraction (UAE) and LC-MS/MS analysis for the forensic determination of COC, BEG, EME, and CET.

VAMS uses an innovative microsampling device, constituted by a hard plastic handle fitted with a detachable tip made of proprietary, porous polymeric material. The tip is capable of absorbing a fixed liquid sample volume (10, 20, or 30 µL), according to its size and independently of sample density or viscosity. After absorption, the specimen is left to dry, and is then ready for storage, shipping or immediate extraction, and analysis [13,14]. The use of fixed-volume dried microsamples offers several advantages over traditional, large-volume biosamples. The water loss slows down or stops most degradation and metabolic processes, thus usually increasing analyte stability and allowing storage at room temperature (RT) for long periods of time [15,16]. The small dried sample can be accommodated in very small storage spaces and be shipped safely and feasibly; it also reduces solvent consumption and is thus cheaper and more sustainable [17,18,19,20,21]. VAMS use in particular is designed to be easily automated, and to sample accurate blood volumes without bias due to variable hematocrit values, which is a major drawback of other microsampling techniques, such as dried blood spots (DBS) on cards [22,23]. Since VAMS was developed specifically for application to whole blood, volume accuracy is granted only for this matrix. Application to other biological fluids requires the study of absorbed volumes under different experimental conditions [19,24].

Although some LC-MS/MS methods can be found in the literature in the last few years, dealing with the analysis of COC, BEG, and EME in human plasma [25,26,27,28,29,30,31], only a few of these also include CET [29,30]. Other papers describe COC, BEG, EME [32], and CET [33,34] determination in capillary or venous whole blood. Some papers deal with the possibility of microsampling for COC and metabolites determination in capillary whole blood, and all of them use some form of DBS for this purpose [33,34,35,36,37]. For obvious reasons, these methods suffer from the general drawbacks of DBS sampling, including the dependence of sampling volume on hematocrit. To the best of our knowledge, no paper has been published until now on the use of VAMS to produce dried microsamples for the simultaneous determination of COC, BEG, EME, and CET both in plasma and whole blood: this approach aims to attain hematocrit-independent microsampling volume, with consequent important benefits in terms of reproducibility and accuracy for blood determinations.

## 2. Results and Discussion

### 2.1. Sampling and Pretreatment Procedure Development

The first step of the study was the testing of VAMS volume accuracy for plasma sampling, followed by the development of a suitable pretreatment procedure. Blood volume accuracy was taken for granted, according to the results of previous reports [38,39,40].

VAMS volume accuracy data at different tip-sample contact times can be found in Table 1. As one can see, mean volume is very accurate (within 1% of the theoretical volume) for all contact times longer than 3 s, and repeatability is also very good (relative standard deviation, RSD < 1.4%). From these results, it appears that VAMS can be confidently used to rapidly, accurately sample plasma as well as whole blood.

After sampling and drying, the analytes were extracted from VAMS tips by solvent extraction. Different solvents and mixtures were tested (methanol, ethanol, acetonitrile, mobile phase, methanol/acetonitrile 50/50, ethanol/methanol 50/50, methanol/acetonitrile/acidic phosphate 10/15/75); although pure solvents provided good extraction yields (> 80%), they also caused a severe matrix effect. Among mixtures, the ternary methanol/acetonitrile/phosphate one provided the best compromise between high recovery (> 86%) and low matrix effect (89% < recovery < 97%) for all analytes and internal standards (ISs). In this case, a very low extraction volume (100 µL) was sufficient to obtain high analyte extraction from both blood and plasma VAMS. Regarding the extraction procedure itself, several workflows were tried: ultrasound-assisted extraction (UAE), vortex-assisted extraction (VAE), and their combinations, applied for different times and at different potencies. Best results were obtained with UAE at 40 kHz for 10 min.

After extraction, the solution was treated as such by dispersive pipette extraction (DPX). DPX is a powerful yet straightforward sample pretreatment technique: It uses very small amounts of chromatographic sorbent, loosely contained in an automatic pipette tip, with procedures similar to those of dispersive solid phase extraction (dSPE). However, DPX sports several advantages over dSPE: It needs minute amounts of sorbent, solvents and sample; and the conditioning and washing steps are often unnecessary, making the procedure more practical and reproducible. Fast, optimal interactions between analyte and sorbent are assured by turbulent mixing, achieved by drawing air into the pipette tip containing the sample and the loose sorbent. For this application, both the conditioning and the washing steps were carefully evaluated and their inclusion in the pretreatment protocol did not lead to performance improvement. For this reason, they were considered unnecessary, so the only steps to be studied were sorbent choice, sample loading, mixing, washing, and elution. After testing different kinds of sorbents (C1, C2, C8, C18, CN, Phenyl), C18 was chosen as the one that gave the best performance. While sample loading is straightforward, with a single drawing of the entire solution into the tip, the three remaining steps were accurately optimized. Sample mixing cycles (1–10) and speed (0.2–1 Hz) were studied, and two cycles at 0.5 Hz gave the best results. Similarly, washing cycles and speed were evaluated and 100 μL of water (2 cycles, 1 Hz) gave the best results. Then, elution solvent (methanol, acetonitrile, phosphate buffer, and mixtures), volume (50–500 µL) and cycles (1–5) were studied. A single cycle at 0.2 Hz with 100 µL of methanol was demonstrated to provide the best combination of extraction yield, sample purification, and speed. By applying the optimized protocol, the entire DPX procedure lasts less than 1 min.

After elution, the sample was injected without any further treatment.

**Figure 2:**
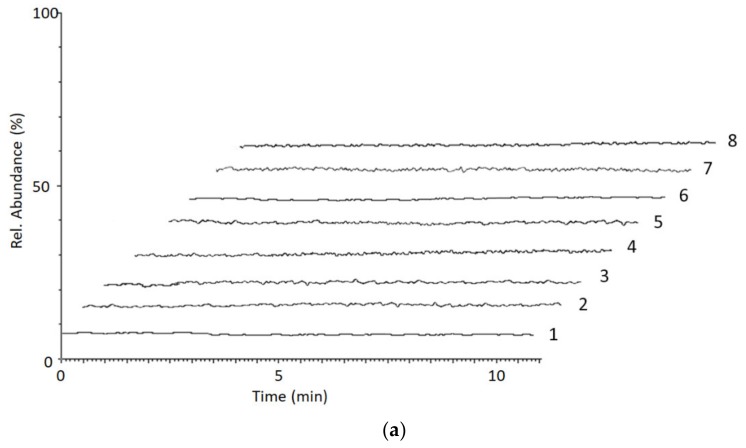

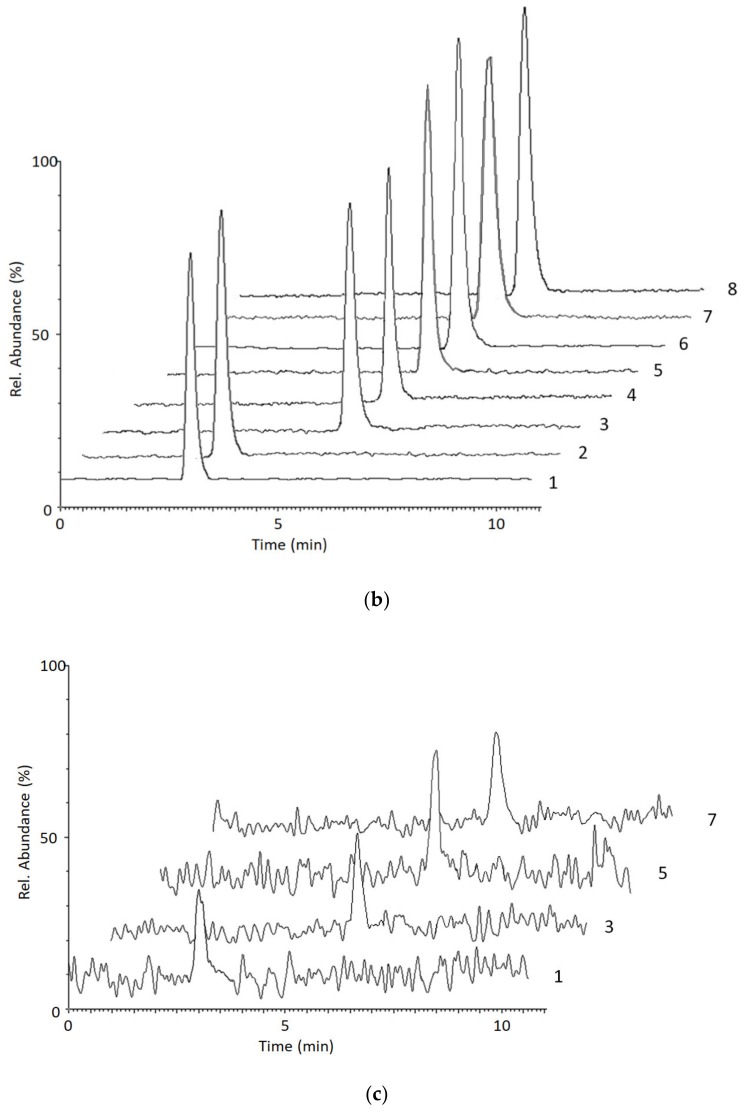
LC-MS/MS chromatograms of (**a**) a blank whole blood VAMS sample; (**b**) a blank plasma VAMS sample spiked with the analytes and the ISs at the concentration of 50 ng/mL; (**c**) a blank plasma VAMS sample spiked with the analytes at the limit of quantitation (LOQ) values: 1—EME; 2—EME-D_3_; 3—BEG; 4—BEG-D_3_; 5—COC; 6—COC-D_3_; 7—CET; 8—CET-D_3_.

Chromatograms of a blank whole blood VAMS sample, pretreated as previously described, are shown in Figure 2a, while chromatograms of a blank VAMS plasma sample spiked with 50 ng/mL of the analytes and the ISs, and with limit of quantitation (LOQ) levels of the analytes, are shown in Figure 2b,c, respectively. As one can see, under these working conditions no endogenous compound interferes with the determination; the analyte peaks are completely separated within 10 min and are satisfactorily symmetrical.

### 2.2. Method Validation

#### 2.2.1. Linearity

Method sensitivity on both blood and plasma VAMS was between 1.0 and 2.5 ng/mL in terms of LOQ, and between 0.3 ng/mL and 0.8 ng/mL in terms of LOD. Method linearity on both matrices was satisfactory, with response factor plot slopes always lower than 1.1 and RSD values lower than 4%. Correlation coefficients (*r*^2^) were always higher than 0.9990 for COC, BEG, EME, and CET (see Table 2).

#### 2.2.2. Process Efficiency, Precision, Matrix Effect and Carry-Over

As can be surmised from Table 3, process efficiency on both VAMS matrices and for all analytes and all concentrations was satisfactory, in the 86%–98% range (92%–96% for the ISs). Precision was also good, producing RSD values always lower than 6.0% (< 5.4% for the ISs). Matrix effect was verified by analyte and IS spiking after sample pretreatment. Complete results are found in Table 3: as one can see, matrix had a really small effect on analyte detectability and quantification; matrix effect magnitude, expressed as percentage response, was in the 89%–97% range for all matrices (91%–96% for the ISs), with slightly better results for COC (> 93%) than for the other analytes. No carry over was observed for both blood VAMS and plasma VAMS (i.e., no signal higher than the background noise at the retention times of the analytes, when injecting a blank solvent after the highest calibrator). Extraction yield comparative assays, performed by adding ISs to whole blood before VAMS sampling and pre-soaking VAMS tips with ISs standard solution, provided satisfactory and overlapping results (RSD < 4.8%), thus demonstrating good applicative suitability of both approaches.

#### 2.2.3. Stability

Analyte stability in dried micromatrices is usually enhanced, due to the lack of water activity. In this study, stability was tested in both plasma and blood VAMS, and in terms of benchtop, autosampler and medium-term stability. Medium-term stability in dried micromatrices stored at RT was compared to fluid matrix stability under freezing (−20°C) conditions.

After 2 months, dried micromatrices stored at RT (20–28°C range) consistently provided higher stability (> 83%) than fluid plasma or blood (> 74%). Benchtop and autosampler stability data were also good for micromatrices (yield > 85%), while macrosamples showed evident signs of degradation (stability < 75%) already after 24 h at RT.

Although COC stability in biosamples is usually reported to be low, even in dried microsamples such as DBS [37], several recent studies have found that the drying step significantly increases stability [33,34,35,36]. It should be noted that the different nature of the VAMS support as opposed to the DBS support (synthetic polymer vs. natural cellulose) could have some effect on analyte stability and extraction as well.

#### 2.2.4. Selectivity

The presence of endogenous interferences was assessed by pretreating and analysing six different blank matrix samples (from healthy volunteers) and comparing the chromatogram of the blanks with the peak area of the LOQ of each analyte, at their respective retention time. Selectivity was considered acceptable if any extraneous peak was ≤ 20% of the response of the LOQ of each analyte.

### 2.3. Analysis of Real Samples and Accuracy

**Figure 3:**
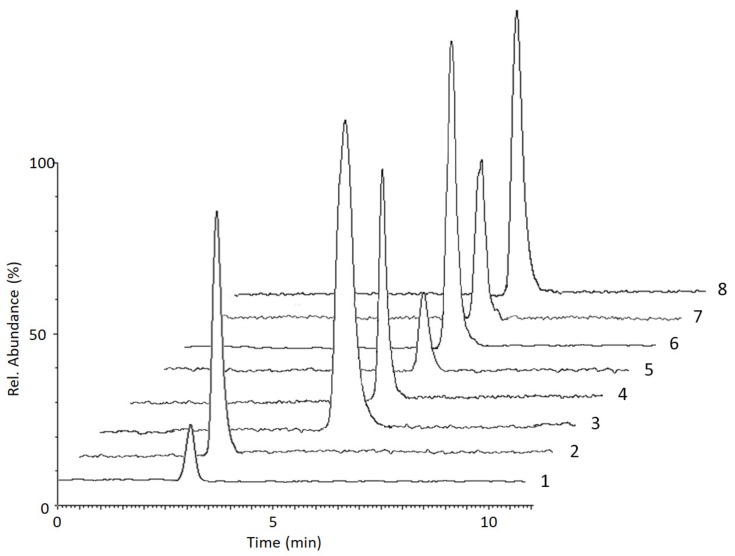
LC-MS/MS chromatogram of a plasma VAMS sample from a COC and ethanol user: 1—EME (12 ng/mL); 2—EME-D_3_ (IS, 50 ng/mL); 3—BEG (196 ng/mL); 4—BEG-D_3_ (IS, 50 ng/mL); 5—COC (18 ng/mL); 6—COC-D_3_ (IS, 50 ng/mL); 7—CET (40 ng/mL); 8—CET-D_3_ (IS, 50 ng/mL).

**Figure 4:**
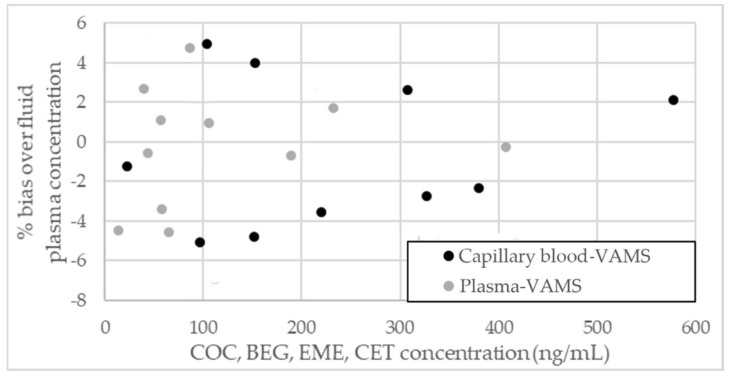
Analyte level comparison between dried micromatrices and fluid plasma.

The methodology was applied to the analysis of macro- and microsamples from individuals who voluntarily admitted to taking COC. To reduce inconsistencies, whenever possible all matrices were simultaneously sampled, either by venipuncture or by fingerpricking in order to carry out a comparison of the results obtained from dried microsamples with those from fluid counterparts and of capillary blood sampled by VAMS with venous blood. In detail: for three subjects, blood was sampled by venipuncture in order to obtain plasma VAMS and to compare it with fluid plasma; for three subjects, both venous blood and capillary blood were concurrently sampled to compare capillary blood VAMS results with venous blood ones (obtained by plasma analysis). As an example, the chromatogram of a plasma VAMS sample from a COC and ethanol abuser is reported in Figure 3. As one can see, sample purification and analyte separation are both satisfactory. Table 4 reports the complete quantitative data obtained from the analysis of real blood and plasma VAMS from six subjects. Usually, raw analytical data from plasma-based matrices can be used as such for quantitation and chemical-clinical correlations [41,42,43,44], while those obtained from blood-based matrices have to be elaborated to account for analyte dilution (correlated to hematocrit), and for red blood cell/plasma partitioning [45,46,47,48,49]. For the samples presented here, no direct hematocrit measurement was carried out: fixed hematocrit values were used, equal to 38% for females and 48% for males. Constant whole blood/plasma concentration ratios of 0.8 for COC, 1.0 for BEG and EME were also applied in all cases, according to literature evidence [50]. Similarly, 1.0 was also used for CET as well, since no information has been found in the literature regarding its partitioning ratio. Both types of correction have already been included in the results of blood-based matrices presented in Table 4 and Figure 4. Of course, when blood concentrations are evaluated, no correction is needed.

Accuracy assays were also performed on the real samples, analysing additional replicates after spiking with different analyte concentrations. Very good accuracy was obtained for both micromatrices, with recovery values always in the 93–104% range.

### 2.4. Comparison to Fluid Blood/Plasma Samples

In order to evaluate the analytical performance of the proposed dried blood and plasma micromatrices, the results obtained from VAMS on real samples (Table 4) were compared to those obtained from fluid plasma analysis by using a fully validated procedure based on solid phase extraction (SPE) coupled to LC-MS/MS analysis. Briefly, the SPE procedure was carried out on Agilent BondElut C8 cartridges (50 mg, 1 mL), previously activated with 2 × 1 mL of methanol and conditioned with 2 × 1 mL of ultrapure water. An aliquot of 400 μL of plasma was loaded onto the cartridge, then sequentially washed with 2 × 1 mL of ultrapure water and with 1 mL of a water/methanol mixture (90:10). The analytes were then eluted with 1 mL of pure methanol, dried under vacuum, re-dissolved with 100 μL of a water/acetonitrile mixture (50:50) containing 0.5% formic acid (FA) and injected into the LC-MS/MS system. As for validation results, extraction yield data were always > 92% (> 94% for ISs); precision results were also satisfactory: RSD values for intraday precision were < 4.0% (< 3.8% for ISs), RSD values for interday precision were < 4.6% (< 4.1% for ISs).

Linear correlation analysis between VAMS and fluid plasma showed that both linearity and slope values were very close to 1, confirming that dried VAMS closely mimic blood or plasma behavior and produce reliable data. Moreover, results obtained from capillary blood sampled by VAMS were consistent with those observed in venous blood obtained by venipuncture. Bias over blood or plasma levels was between −4.5% and +4.8% for plasma VAMS and between −5.1% and +4.9% for blood VAMS (see Figure 4, where plasma VAMS and calculated plasma concentrations from capillary blood VAMS were compared with fluid plasma results).

Previous studies performed on DBS observed a lower correlation between capillary blood sampled by VAMS and venous blood. This could be due to the higher overall result variability obtained on DBS, which in turn may be due to several factors, including volume of blood spotted onto the card, blood hematocrit and lack of spot homogeneity [37,51].

Slightly better performance of plasma-VAMS than blood-VAMS could be due to the lack of particulate and thus of hematocrit effect. Both dried matrices are surely more practical and more easily handled than fluid blood plasma. Among micromatrices, blood-VAMS is more practical than plasma-VAMS, since it can be obtained with a minimally invasive fingerpick and does not require any sample manipulation prior to sampling. However, blood-based VAMS has the added complication of hematocrit and its effect on analyte concentration, as mentioned above (although the effect of hematocrit on analyte concentration is usually very small for COC and most of its metabolites).

## 3. Materials and Methods

### 3.1. Chemicals and Standard Solutions

Certified methanolic stock solutions of COC, BEG, EME and CET (1 mg/mL) and of COC-D_3_, BEG-D_3_, EME-D_3_, and CET-D_3_ (100 μg/mL), used as ISs; acetonitrile, methanol, potassium phosphate monobasic, formic acid (FA)—all reagents for mass spectrometry—and other solvents used for sample preparation (all analytical grade) were purchased from Sigma Aldrich Italy (Milan, Italy). Ultrapure water (18.2 MΩ∙cm) was obtained by means of a Milli-Q apparatus from Merck Millipore (Darmstadt, Germany). Analyte standard solutions were prepared daily by dilution with a water/acetonitrile mixture (50:50) containing 0.5% FA. All solutions were stored protected from light in amber glass vials certified for mass spectrometry from Waters (Milford, MA, USA).

### 3.2. LC-MS/MS Instrumentation and Conditions

LC-MS/MS analysis was performed on a Waters Alliance e2695 chromatographic system with autosampler coupled to a Waters Micromass Quattro Micro triple-quadrupole mass spectrometer equipped with an electrospray ion source (ESI). Data processing was performed using Waters MassLynx 4.1 software. Separations were obtained on a reverse phase XBridge BEH C8 (50 × 3.0 mm; 2.5 µm) column from Waters, maintained at room temperature and equipped with a guard column (C8, 4 × 3 mm). The mobile phase was a mixture of 0.5% aqueous formic acid (component A) and 0.5% formic acid in acetonitrile (component B), at a constant rate of 0.3 mL/min and under the following composition gradient: 10% B from 0 to 3 min, then B increased to 90% at 7 min, hold 90% B for 3 min, 10% B at 13 min, and re-equilibrate at 10% B for 2 min. The total run time was 15 min, including column re-equilibration. The injection volume was 10 μL.

Multiple reaction monitoring (MRM) transitions were used, acquiring in positive ionization mode (ESI+) and exploiting two different exclusive transitions for each analyte: the most abundant one for quantitative purposes, the second one for identity confirmation. The optimized parameters were as follows: ion source voltage, 4.5 kV; ion source temperature, 140 °C; desolvation temperature, 300 °C; desolvation gas flow, 550 L/h; (nitrogen as the desolvation gas, argon as the collision gas). The precursor ions and the product ions, with dwell time, cone voltage and collision energy, were optimised and are shown in Table 5.

### 3.3. Compliance with Ethical Standards

Blood samples, used as blank matrices, were obtained from drug-free healthy volunteers. Real blood samples were from volunteers who declared they had used COC, and possibly ethanol, or from subjects who had obtained a positive result in COC drug tests. All samples, including fingerprick ones, had already been collected for other forensic needs. All subjects provided informed consent prior to their participation.

### 3.4. Microsampling: VAMS

Mitra^®^ VAMS microsamplers (20 µL) were provided by Neoteryx (Torrance, CA, USA) and include a polypropylene handle (about 4 cm long) topped with a small tip (about 2-mm diameter) of a proprietary polymeric porous material.

Plasma VAMS volume accuracy and precision were tested by weighing complete, unused devices, then carrying out the sampling procedure (see below) for different contact times (1–20 s) and weighing the device again immediately. The difference between device weight before and after sampling was used to calculate the plasma volume sampled, using a fixed, conventional plasma density of 1025 kg/m^3^. The procedure was repeated 6 times for each contact time, in order to calculate RSD% values.

Blank or blank spiked blood VAMS were obtained by drawing a few milliliters of blood from volunteers into vials without any anticoagulant, then spiking it with the analytes and the ISs (if needed) and sampled within few minutes in order to avoid both coagulation and analyte degradation by blood esterases. The tip of a VAMS device was then immediately put into contact with the blood surface and kept there, at a 45° angle, for 2 s. The device was dried at RT for 1 h and stored at RT, in the dark for 2 months at most, in a dedicated clamshell in order to avoid contact with any surface. Clamshells were stored in a sealed polyethylene bag containing desiccant. The microsampler tip was detached from the handle and subjected to UAE at 40 kHz for 20 min in 100 µL of methanol/acetonitrile/50 mM, pH 3.0 phosphate buffer (10/15/75, *v*/*v*/*v*). The resulting solution was then subjected to DPX pretreatment.

Real blood VAMS were obtained by pricking a fingertip with a disposable, sterile steel needle. The tip of a VAMS device, previously soaked with an IS solution and dried, was then put into contact with the surface of the blood droplet and kept there, at a 45° angle, for 2 s. All subsequent steps are identical to those of blank blood VAMS.

Blank, blank spiked or real plasma VAMS were obtained and handled in the same way as blank blood VAMS, except the blood was centrifuged and the resulting supernatant plasma separated. Then, the plasma was spiked (if needed) and the tip of a VAMS device was put into contact with its surface.

ISs addition mode and extraction performance assays (*n* = 6) were carried out by comparing pooled blood fortification (as in blank spiked samples) with VAMS tip pre-soaking (as in real capillary blood VAMS sampling) in order to exclude any bias in terms of actual spiked concentration and extraction efficiency.

### 3.5. Microsample Pretreatment: DPX

DPX devices (C18, 100 µL) were obtained from DPX Technologies (Columbia, SC, USA). The 100 µL extract obtained from VAMS pretreatment was loaded onto the pipette tip, then mixed (2 cycles, 0.5 Hz) and finally discarded. Afterwards, the sorbent was washed with 100 μL of water (2 cycles, 1 Hz). The analytes were eluted using 100 μL of methanol (1 cycle, 0.2 Hz).

The eluate was directly injected into the LC-MS/MS system.

### 3.6. Method Validation

The analytical method was validated according to European Medicines Agency (EMA), Food and Drug Administration (FDA), World Anti-Doping Agency (WADA) and International Conference on Harmonization (ICH) guidelines [52,53,54,55]. The tested parameters were linearity (including limits of detection and limit of quantitation), extraction yield, precision, matrix effect, stability, and accuracy.

Blood and plasma samples from volunteers not using COC were spiked with the analytes at seven different concentrations, containing the ISs at a constant concentration, subjected to VAMS and DPX and injected into the LC-MS/MS system. The analysis was carried out in triplicate for each concentration. The obtained analyte/IS peak area ratios were plotted against the corresponding concentrations (expressed as ng/mL) and the calibration curves were obtained by means of the least-square method. A 1/x^2^ weighing factor was applied. LOQ was calculated as the analyte concentration which gave rise to peaks ≥ five times the analyte response of a blank sample, as long as the percentage relative standard deviation (RSD%) of peak area ratio was lower than 20%. Linearity was evaluated by means of response factor plots.

Process efficiency was evaluated by repeatedly subjecting blank samples spiked with analyte standard solutions at three different, known concentrations (corresponding to a low, an intermediate and a high value of the linearity range) to the previously described procedure. The obtained analyte peak areas were compared with those obtained by injecting standard solutions at the same theoretical concentrations in order to calculate process efficiency. Acceptability criterion: efficiency > 80%. Precision assays were carried out on the same microsamples; six replicates were analyzed in the same day to obtain intraday precision and over six different days to obtain interday precision, expressed as RSD%. Acceptability criteria: RSD < 10% intraday (< 20% for the LOQ level), RSD < 15% interday (< 20% for the LOQ level).

IS-corrected matrix effect was evaluated on blank samples extracted from 6 different sources, fortified post-extraction by adding known amounts of the analytes and then analyzed by LC-MS/MS. Mean analyte/IS peak area ratios of each extract were compared with analyte/IS peak area ratios from standard solutions and the resulting percentage responses were calculated. Acceptability criterion: response = 100 ± 15%.

Carry-over was assessed by injection of blank solvent (50:50 water/acetonitrile mixture containing 0.5% FA) after the highest calibrator (*n* = 3). The absence of carry-over was accepted if the response at the retention time of the analytes was less than 20% of the LLOQ values.

To test analyte stability, VAMS samples were obtained from venous blood spiked with the analytes at two concentration levels (high and low concentrations with respect to the calibration curve), then stored at RT, protected from light, in sealed polyethylene bags containing desiccant for 2 months. At regular intervals (1 week), microsamples were pretreated and analyzed in triplicate. The measured analyte concentrations were compared to those of the same samples extracted and analyzed immediately after sampling and drying. The stability values thus obtained were also compared to those of blood or plasma macrosamples stored at −20°C. For autosampler stability of processed VAMS, samples spiked at the same two concentration levels were freshly pretreated in triplicate and the extracts were stored in the autosampler at RT for 24 h before re-analysis, while for VAMS bench-top stability, spiked VAMS samples were stored for 3 days at room temperature without using neither polyethylene bags nor desiccant. Samples were considered stable when the bias from nominal concentrations was within ± 15%.

Recovery assays were carried out in order to evaluate method accuracy: standard solutions containing known amounts of the analytes (corresponding to a low, an intermediate and a high value of the respective calibration curves) and fixed amounts of the ISs were absorbed by soaking blank VAMS tips in standard solutions prepared in a water/acetonitrile mixture (50:50) containing 0.5% FA, then dried and the usual plasma and capillary blood VAMS procedure was carried out on real samples. The obtained additional fortified real samples were then analyzed, and the recovery of spiked analytes was calculated by comparison to the real samples without spiking. Acceptability criterion: recovery > 80%.

## 4. Conclusions

In conclusion, this study is a proof-of-concept that confirms the versatility of blood and plasma dried microsampling by VAMS and its suitability for most forensic purposes. In particular, VAMS allows the development of fast, accurate and precise analytical methods that require minimal, feasible sample pretreatment. Moreover, blood VAMS sampling is usually not sensitive to hematocrit variability, a parameter that affects most other microsampling techniques to different degrees; on the other hand, extraction performance from VAMS could, at least theoretically, be affected by hematocrit. As regards plasma VAMS analysis, despite the fact that the analytical workflow requires additional whole blood centrifugation and plasma separation steps, it holds all the logistical and analytical advantages previously described, making it an attractive alternative to usual plasma sampling.

Further assays are now in progress to extend the detectability of COC use by VAMS and LC-MS/MS, with the inclusion of other long-term metabolites, and to apply the workflow to other drugs of abuse. Method automation, self-testing, and at-home testing are other fields of investigation that portend important developments for the application of VAMS to numerous settings and contexts, including forensic analysis.

## Figures and Tables

**Table 1:** Plasma volumetric absorptive microsampling (VAMS) volume accuracy and precision results.

Tip Contact Time (s)	Sampling Volume (µL)	Volume Accuracy (% of Theoretical Value)	Volume Precision (Relative Standard Deviation, RSD%)
1	17.06	85.3	3.0
2	18.46	92.3	2.2
3	19.14	95.7	1.1
5	20.02	100.1	1.3
10	19.90	99.5	0.9
15	19.98	99.9	0.9
20	20.20	101.0	1.0

**Table 2:** Linearity, LOQ, LOD on spiked matrices.

Analyte	Matrix	Linearity Range (ng/mL)	Linearity Equation ^1^	*r* ^2^	LOQ (ng/mL)	LOD (ng/mL)
COC	Blood VAMS	2.0–500	y = 0.446x + 0.003	0.9995	2.0	0.6
	Plasma VAMS	2.0–500	y = 0.121x + 0.005	0.9997	2.0	0.6
BEG	Blood VAMS	1.0–500	y = 0.861x + 0.003	0.9992	1.0	0.3
	Plasma VAMS	1.0–500	y = 0.224x + 0.006	0.9996	1.0	0.3
EME	Blood VAMS	2.5–500	y = 0.030x + 0.004	0.9993	2.5	0.8
	Plasma VAMS	2.5–500	y = 0.083x + 0.008	0.9995	2.5	0.8
CET	Blood VAMS	2.0–500	y = 0.428x + 0.004	0.9991	2.0	0.6
	Plasma VAMS	2.0–500	y = 0.141x + 0.008	0.9994	2.0	0.6

^1^*y* = analyte/IS area ratio, dimensionless; *x* = analyte concentration, ng/mL.

**Table 3:** Process efficiency, precision and IS-corrected matrix effect assay results.

**Analyte**	**Concentration Level (ng/mL)**	**Matrix**	**Precision, RSD% ^1^**		**Process Efficiency** **± SD, %**	**Matrix Effect** **± SD, %**
			**Intraday**	**Interday**		
COC	2.0	Blood VAMS	5.2	5.3	91 ± 3	93 ± 2
		Plasma VAMS	4.9	5.0	93 ± 2	94 ± 3
	50	Blood VAMS	5.0	5.2	96 ± 3	93 ± 2
		Plasma VAMS	4.6	4.9	96 ± 4	95 ± 1
	500	Blood VAMS	4.8	5.1	95 ± 1	95 ± 4
		Plasma VAMS	4.3	4.7	98 ± 2	97 ± 2
BEG	1.0	Blood VAMS	5.4	5.4	88 ± 3	89 ± 1
		Plasma VAMS	5.1	5.4	90 ± 4	91 ± 4
	50	Blood VAMS	5.2	5.3	91 ± 2	89 ± 3
		Plasma VAMS	4.9	5.0	94 ± 1	92 ± 4
	500	Blood VAMS	4.9	5.3	93 ± 3	91 ± 1
		Plasma VAMS	4.5	4.8	94 ± 2	91 ± 2
EME	2.5	Blood VAMS	5.3	5.8	87 ± 4	90 ± 4
		Plasma VAMS	5.3	5.6	91 ± 3	91 ± 2
	50	Blood VAMS	5.6	5.6	92 ± 2	91 ± 3
		Plasma VAMS	5.1	5.4	93 ± 3	93 ± 3
	500	Blood VAMS	5.0	5.2	92 ± 2	92 ± 3
		Plasma VAMS	4.8	5.1	95 ± 2	93 ± 4
CET	2.0	Blood VAMS	5.3	5.9	86 ± 3	89 ± 1
		Plasma VAMS	5.1	5.6	89 ± 3	92 ± 2
	50	Blood VAMS	4.9	5.4	89 ± 2	91 ± 2
		Plasma VAMS	4.9	5.2	94 ± 2	93 ± 4
	500	Blood VAMS	5.0	5.4	91 ± 3	91 ± 1
		Plasma VAMS	4.6	4.9	94 ± 2	94 ± 3
COC-D_3_	50	Blood VAMS	4.8	4.9	95 ± 2	94 ± 1
		Plasma VAMS	4.3	4.5	96 ± 3	96 ± 1
BEG-D_3_	50	Blood VAMS	5.0	5.0	92 ± 2	91 ± 2
		Plasma VAMS	4.6	4.7	95 ± 2	93 ± 2
EME-D_3_	50	Blood VAMS	5.2	5.3	93 ± 2	94 ± 3
		Plasma VAMS	4.7	5.0	95 ± 2	95 ± 2
CET-D_3_	50	Blood VAMS	4.5	4.7	92 ± 3	94 ± 2
		Plasma VAMS	4.6	4.9	95 ± 1	96 ± 2

^1^*n* = 6.

**Table 4:** Analyte level results in real VAMS samples.

Subject	Matrix	Concentration Found ± SD (ng/mL) ^1^			
		COC	BEG	EME	CET
1	Capillary blood VAMS	216 ± 8	584 ± 12	156 ± 6	/
2	Capillary blood VAMS	153 ± 4	376 ±9	94 ± 5	28 ± 5
3	Capillary blood VAMS	322 ± 9	312 ± 8	106 ± 5	/
4	Plasma VAMS	108 ± 4	407 ± 10	88 ± 4	57 ± 6
5	Plasma VAMS	19 ± 3	193 ± 4	13 ± 2	39 ± 5
6	Plasma VAMS	63 ± 3	234 ± 8	54 ± 3	/

^1^*n* = 3.

**Table 5:** Multiple reaction monitoring (MRM) transitions and compound-specific MS parameters.

Analyte	Q1 (*m/z*)	Q3 (*m/z*)^1^	Dwell Time (ms)	Cone Voltage (V)	Collision Energy (eV)
COC	304.27	82.1	200	40	40
		*182.0*			*35*
BEG	290.16	82.1	200	60	35
		*168.1*			*25*
EME	200.13	182.0	200	35	30
		*82.1*			*25*
CET	318.24	196.1	200	60	35
		*150.1*			*30*
COC-D_3_	307.26	185.1	200	60	30
BEG-D_3_	293.25	85.1	200	50	30
EME-D_3_	203.25	185.1	200	60	25
CET-D_3_	321.26	199.0	200	60	30

^1^ In italics, qualifier ions
